# Morphospace exploration reveals divergent fitness optima between plants and pollinators

**DOI:** 10.1371/journal.pone.0213029

**Published:** 2019-03-13

**Authors:** Foen Peng, Eric O. Campos, Joseph Garret Sullivan, Nathan Berry, Bo Bin Song, Thomas L. Daniel, H. D. Bradshaw

**Affiliations:** Department of Biology, University of Washington, Seattle, Washington, United States of America; Universidade Federal de Uberlândia, BRAZIL

## Abstract

The obligate mutualism and exquisite specificity of many plant-pollinator interactions lead to the expectation that flower phenotypes (e.g., corolla tube length) and corresponding pollinator traits (e.g., hawkmoth proboscis length) are congruent as a result of coevolution by natural selection. However, the effect of variation in flower morphology on the fitness of plants and their pollinators has not been quantified systematically. In this study, we employed the theoretical morphospace paradigm using a combination of 3D printing, electronic sensing, and machine vision technologies to determine the influence of two flower morphological features (corolla curvature and nectary diameter) on the fitness of both parties: the artificial flower and its hawkmoth pollinator. Contrary to the expectation that the same flower morphology maximizes the fitness of both plant and pollinator, we found that the two parties have divergent optima for corolla curvature, with non-overlapping fitness peaks in flower morphospace. The divergent fitness optima between plants and pollinators could lead to evolutionary diversification in both groups.

## 1. Introduction

Plant-pollinator interactions are considered to be classical examples of mutualism [1]. Pollinators provide plants with pollen transport services leading directly to offspring production, while plants provide pollinators with many types of rewards, such as energy-rich nectar [2], shelter [3], or thermoregulation[4–5], that enhance the survival, growth, and ultimately reproductive output. As a result, flower traits are expected to be congruent with the corresponding phenotypes of their pollinators, particularly in obligate mutualisms where each party is completely dependent on the other for survival and reproduction. A striking example of this congruence is Darwin’s famous prediction of the existence of a hawkmoth species with a long proboscis to match the extraordinarily long nectar spur of a Malagasy orchid [6]. Such precise, predictable phenotypic matching between flower and pollinator morphologies suggests that flower shape is optimized by coevolution to maximize the fitness of both the plant and the pollinator, but this hypothesis has never been explicitly tested in a comprehensive way, partly because of the empirical challenges in manipulating flower morphologies and estimating fitness. Given the phenomenal diversity of flower morphologies in nature, an approach which allows quantifying and manipulating morphological variation along multiple axes is necessary to comprehensively map the fitness landscape in plant-pollinator interactions.

Raup [7] proposed a general framework for discovering the functional consequences of morphological variation: the theoretical morphospace paradigm. This approach explores “n-dimensional geometric hyperspaces produced by systematically varying the parameter values of a geometric form” [8]. Raup devised a simple and elegant way to describe variation in the shape of mollusk shells using a 3-parameter equation, then tested the hydrodynamic performance of artificial shells fabricated to sample a wide range of the total 3-dimensional morphospace. Because the bounds of this theoretical morphospace are not constrained by naturally existing forms, it enables unbiased study over all the theoretically possible forms, which includes those that never have occurred in nature.

**Fig 1:**
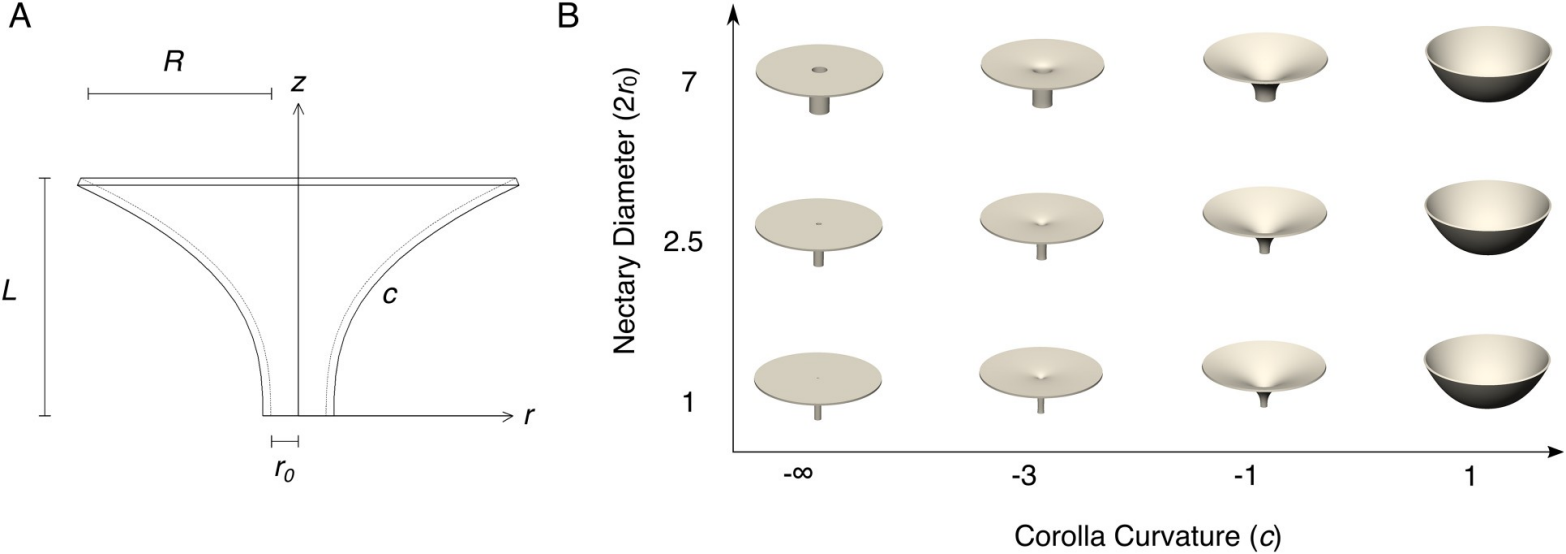
Design of artificial flowers. (A). Side view of a flower, showing the four parameters in the equation. All parameters (except for *c*, which is dimensionless) are in millimeters. (B). 3D rendering of some representative flowers with variation in nectary diameter and corolla curvature. The corolla tube length *L* is fixed at 20 mm and the overall diameter 2(*R* + *r*_*0*_) is fixed at 55 mm.

In the spirit of the theoretical morphospace paradigm [8], we previously defined flower morphology with a 4-parameter equation (Fig 1A), fabricated the flower models with a 3D printer (Fig 1B), and experimentally tested a hawkmoth pollinator’s ability to exploit the flower’s nectar. We showed that both flower curvature and nectary diameter influence the hawkmoth pollinator’s performance [9].

In this study, we explored the fitness landscapes along two flower shape axes (corolla curvature and nectary diameter). Contrary to the expectation that a single flower morphology maximizes the fitness of both plant and pollinator, we found that the two parties have divergent optima, with non-overlapping fitness peaks in flower morphospace.

## 2. Materials and methods

Pollinator visitation experiments were carried out with lab-reared hawkmoths (2.1) and 3D-printed flowers (2.2) containing an artificial nectar solution of 20% w/v sucrose (hereafter, nectar). We performed the visitation experiments in two stages. In the first stage, we focused on the *quantity* of pollinator visits and explored the fitness landscapes along two flower shape axes (corolla curvature and nectary diameter) (2.3). In the second stage, we explicitly measured the *quality* of each visit, in terms of the efficiency of nectar acquisition by the pollinator (a correlate of pollinator fitness) and the number of pollinator contacts with the plant’s reproductive parts (a proxy for plant fitness), in a critical region of flower morphospace (2.4).

### 2.1 Pollinators

Individual *Manduca sexta* hawkmoths were obtained from a colony maintained by the Department of Biology at the University of Washington. Hawkmoths were flower-naïve and unfed for 1–3 days post-eclosion. Each hawkmoth’s sex and proboscis length were recorded before it was used in experiments, and the effects of each of these variables on pollinator performance tested.

### 2.2 Fabrication of artificial flowers

The artificial flowers used in this study were fabricated in rigid, white polyvinyl chloride (PVC) plastic using a 3D printer by methods previously described [9]. Flower models were designed in SolidWorks (Dassault Systèmes SolidWorks Corporation, Waltham, Massachusetts, USA) based on an equation with four flower shape parameters: corolla curvature, nectary radius, flower length, and corolla radius. In a manner similar to that of Raup [7], we used a parametric equation (Eq 1) for a surface generating curve [9]:

$$z(r)=L{\left(\frac{r-r_{0}}{R}\right)}^{e^{c}},$$ (1)

where *z* represents the longitudinal axis of the flower model, *r* represents the radial distance of the corolla from the central *z*-axis, *c* is a curvature parameter determining the lateral profile of the corolla (note that the *c* parameter in our equation is not equivalent to the definition of curvature in mathematics), *r*_*0*_ is the nectary radius, *L* is the flower length, and *R* is the lateral extent of the corolla from edge of the nectary to the outer lip of the flower (*i*.*e*., the full radius from the central z-axis is equal to *r*_*0*_+ *R*) (Fig 1A). The unit of all parameters (except *c*, which is dimensionless) is millimeter (mm). This curve is rotated about the z-axis and given a 1 mm thickness to create a 3D model.

### 2.3 First stage experiment to measure the *quantity* of pollinator visits

#### 2.3.1 Experimental apparatus and flight arena

Artificial flowers were arranged in a 6 by 6 square array with flower centers spaced at 30.5 cm (S1 Fig). Modular T-slot extruded aluminum (80/20 Inc.) was used as the structural support for the flower array. Each 36-flower array was populated with 6 distinct flower morphologies, present at equal frequencies. Flower positions were randomized before each foraging trial. Each artificial flower’s nectar reservoir was filled with 20 μL of nectar solution. The flower array was located inside a flight chamber (2.4 m width x 4.0 m length x 2.5 m height). Two dim white LED lights illuminated the arena from above, calibrated to a combined illuminance of 0.1 lux at flower level to simulate moonlight conditions. The flight chamber was also illuminated from above with a single infrared light (Magnalight LEDLB-16E-IR; 790–880 nm flat emission peak, modified by removing the light’s focusing lenses to create even, diffuse lighting), invisible to *M*. *sexta*, to allow video recording of the hawkmoth’s foraging trajectory (S1 Fig).

Each artificial flower was held in the array by a 3D-printed bracket that contained an infrared (IR) emitter-detector pair, creating an infrared beam sensor. When a hawkmoth’s proboscis entered the nectar reservoir of any artificial flower, the infrared beam was broken, and this event was recorded through a data acquisition system consisting of an Arduino microcontroller (Sparkfun: DEV-11021) and a laptop computer (S1 Fig). Data collection ended when all six flowers of any single morph were exploited. An infrared-sensitive video camera was mounted above the flower array facing straight down, to capture the hawkmoth’s flight trajectory as it foraged on the artificial flowers.

The air temperature of the flight arena was kept at approximately 24°C. To stimulate hawkmoth foraging, 5 μL of a 7-component synthetic scent mixture [9] was placed in the flight chamber 5 min before the experiment began. The synthetic scent mixture mimics the scents emitted by the flower of the hawkmoth-pollinated plant *Datura wrightii*.

#### 2.3.2 Experimental treatments and sample size

A foraging trial ended when the hawkmoth left the flower array and no longer visited flowers. Foraging trials typically lasted from 4 to 12 minutes. No hawkmoth participated in more than one foraging trial. After each trial, emptied flowers were counted, empty nectar tubes were replaced with fresh pre-filled nectar tubes, and the positions of the flower morphs were re-randomized in preparation for the next foraging trial.

Hawkmoths were tested with various combinations of corolla curvature (*c*: −∞, -4, -3, -2, -1, 0, 0.375 and 1) and nectary diameter (2*r*_*0*_: 1, 1.75, 2.5, 3.25, 5, and 7 mm) (Fig 1B). In addition to flower exploitation data in the form of counts of emptied flower morphs, the number of visits paid to each flower morph was counted by analyzing the video recording of each of 125 foraging trials (S1 Table).

#### 2.3.3 Data processing and statistical analysis

Two-way analysis of variance (ANOVA) was used to test for differences in flower visitation frequency and hawkmoth foraging success rate among flower morphologies varying in corolla curvature and nectary diameter. Statistical analyses were performed in R version 2.3.2. Flower visitation frequency is an estimate of plant fitness, calculated as the number of hawkmoth visits to each flower morphology per trial. Hawkmoth foraging success rate is an estimate of pollinator fitness on each flower morphology, calculated as the number of emptied flowers per morphology per trial divided by the number of visits to that morphology per trial. Flower morphologies which received 0 visits in a trial were excluded from foraging success rate analysis.

### 2.4 Second stage experiments focused on the *quality* of pollinator visits

#### 2.4.1 Experimental apparatus and flight arena

**Fig 2:**
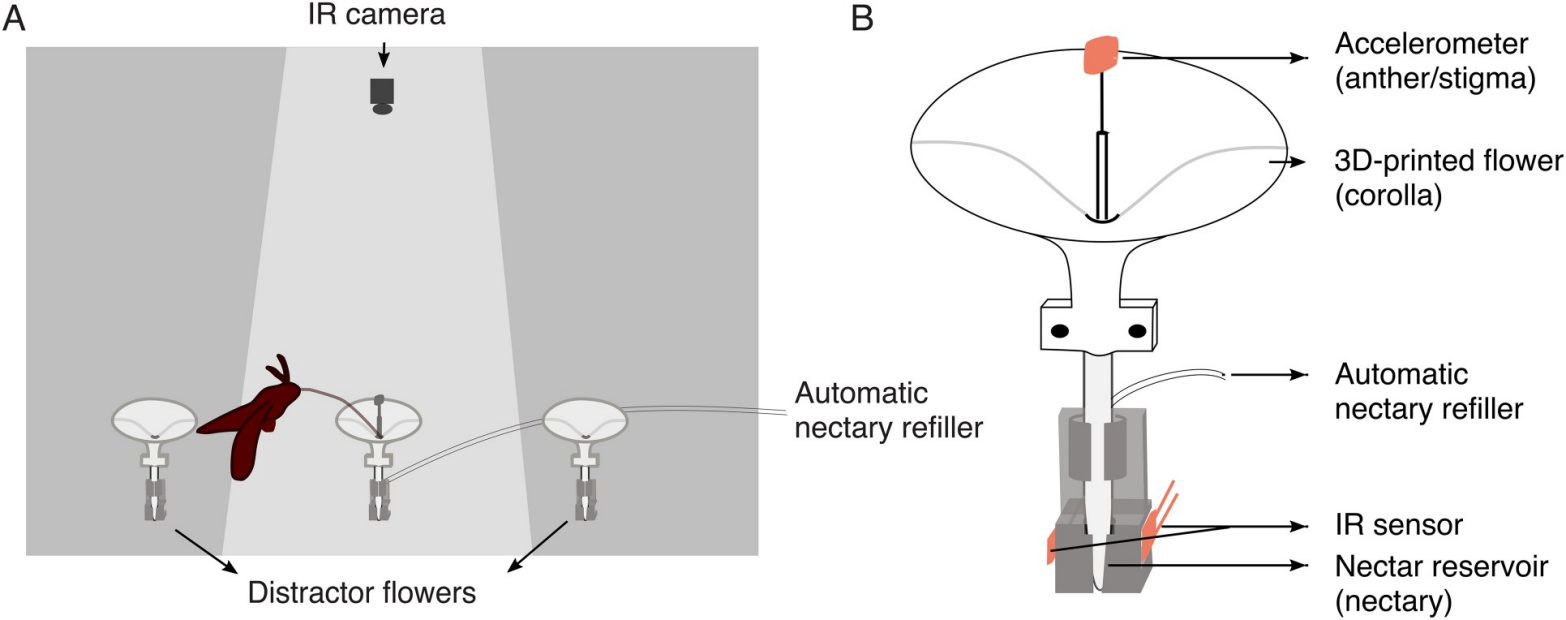
Second stage experimental setup. (A). Hawkmoth flight chamber. Only the center flower is instrumented and supplied with nectar. The two flowers on the sides are distractors. (B). Instrumented flower model, which is an enlarged view of the center flower in (A).

In the second stage experiments, we focused on flower-hawkmoth interaction in the region of flower morphospace identified in the first stage as most sensitive to variation in corolla curvature (*c*) and nectary diameter (2*r*_*0*_). Individual hawkmoths were presented with only one nectar-bearing flower in the flight arena, to examine explicitly the effectiveness of each pollinator visitation event. The experimental flower apparatus consisted of a 3D-printed flower model, a micro-accelerometer assemblage to record the physical contact between the pollinator and the “reproductive parts” of the flower, and a nectary assemblage to provide nectar and detect nectar level changes (Fig 2B).

A small (4 x 4 x 1.45 mm) 3-axis accelerometer (ADXL335, SparkFun, Niwot, Colorado, USA) was used as a proxy for the flower’s reproductive organs (*i*.*e*., anthers and stigma). The accelerometer was solder-connected with ultra-thin silver wire (0.14mm) and supported on a 20mm long thin stainless steel wire (0.13mm), mimicking the filament and style to support the anthers and stigma in real flowers. The other end of the steel wire was inserted into a rigid stainless steel tube to fix the free vibrating length of the wire, so that the natural frequency of the wire (about 18.4 Hz) did not change during the experiment. The accelerometer was positioned in the center of the corolla, 10 mm above the flower top plane.

A 25 mm long rigid plastic tube (inside diameter = 3.18 mm) connected the printed flower and the nectary assemblage. The distance from the plane of the flower top to the bottom of nectar reservoir was 70mm, which is shorter than the average proboscis length of *M*. *sexta* (82.5 mm, N = 58). The same infrared emitter-detector pair as in the first stage experiments was installed on the side of nectar base, allowing infrared light to pass through the bottom of the nectar reservoir to record nectar level changes. We constructed an automatic nectary refiller using a micro-injector pump, controlled by an Arduino Uno microcontroller, to push a nectar-filled 500 μL Hamilton syringe (Part No. 80865) with a stepper motor (adapted from [10]), to automatically replenish 20 μL of nectar during the trial. The micro-injector had enough capacity to refill the nectar reservoir 25 times, and was located outside the flight chamber.

The experimental flower apparatus was affixed to the floor of a flight chamber. Two distractor flowers (morphology: *r*_*0*_ = 1 mm, *c* = -3, *L* = 30 mm, *R* = 24 mm), which had no accelerometer assemblage or nectar supply, were placed 25 cm on either side of the experimental flower. The distractor flowers were used to distract the hawkmoth from the experimental flower, so that the nectar in the experimental flower could be replenished after the hawkmoth left. A webcam (C170, Logitech, Lausanne, Switzerland) was affixed to the ceiling of the flight chamber, facing vertically down at the experimental flower. The videos were taken at a frequency of 5 frames/sec. The lighting and scent conditions in the flight chamber were identical with the first stage experiments (Fig 2A).

#### 2.4.2 Experimental treatments and sample size

Moths were presented with one instrumented artificial flower per trial. We tested four different corolla curvatures (*c*: −∞, -3, -1, and 1; fixing other floral shape parameters at 2*r*_*0*_ = 3 mm; *L* = 30 mm; *R* = 23.5 mm). A 3 mm nectary diameter was used to account for the presence of the artificial anther/stigma (the diameter of the wiring is about 0.5 mm) partially occluding the corolla tube, leaving an open diameter of 2.5 mm. This diameter is consistent with measured nectary diameter values for hawkmoth-pollinated flowers such as *Petunia axillaris* and *Datura wrightii* which have nectary diameters ranging from 1 to 2.5 mm [11]. Accelerometer and infrared nectar sensor data were collected through an Arduino Uno microcontroller at a frequency of 1 kHz. A foraging trial ended when either: 1) the nectar had been emptied 25 times (*i*.*e*., reached the capacity of the micro-injector); or, 2) four minutes had passed since the last visit to the instrumented flower. For each flower morph, we collected data from 14–15 hawkmoths. The total number of hawkmoths over all trials was 58.

#### 2.4.3 Data processing and statistical analysis

The videos taken by the webcam were analyzed with a customized Python program (Github repository: https://github.com/foenpeng/Controller-of-experiment-platform.git). Because the instrumented flower and webcam were both static, the hawkmoth’s location in each frame could be obtained by frame subtraction. A reference frame was taken before a hawkmoth started foraging. Every new frame was subtracted from this reference frame. The centroid of the largest contour in every subtracted frame was used as an estimate of the hawkmoth’s position. The coordinates of the centroid were compared with a 125 mm radius circle (about the total length of a hawkmoth’s body plus a fully extended proboscis) centered on the experimental flower to determine the presence/absence of the hawkmoth pollinator. A pollinator visit was recorded whenever the hawkmoth centroid was inside the 125 mm radius.

We used the amount of acquired energy (from nectar) divided by the total time spent within 125 mm of the instrumented flower during each visit to represent the pollinator’s rate of energy gain per flower visit, which is a correlate of hawkmoth fitness [12]. The total amount of time that each hawkmoth spent during each visit to the instrumented flower was calculated from the video tracking data. The amount of energy acquired is fixed for every visit: the hawkmoth always consumes all of the 20 μL of 20% sucrose nectar in the container if it reaches nectar, which contains 0.004 g sucrose (16.2 kJ/g), providing a maximum of 64.8 J of chemical energy assuming that all energy ingested is available; if the hawkmoth fails to reach nectar, it obtains 0 J energy. The rate of energy expenditure is minute relative to the rate of energy gain (2–3% of acquired energy [12]).

As a proxy for the plant’s fitness, the total number of “hits” on the accelerometer (representing the anthers and stigma of a real flower) during each hawkmoth visit was inferred from the accelerometer data. The analog signal readout from the accelerometer was calibrated using gravitational acceleration (*g* = 9.8 m s^-2^) when the accelerometer was in a static state. The total acceleration was calculated as the square root of the sum of squares of acceleration in each of the three axes (x, y, z). A “hit” was identified with a peak detection algorithm (Python peaktutils package), with total acceleration greater than 3 *g* counted as a hit.

The plant’s and pollinator’s fitness with respect to flower morphology (corolla curvature) variation were analyzed by ANOVA.

Variation in hawkmoth morphology (proboscis length) and its impact on the plant’s and pollinator’s fitness was also examined. We performed a Student’s t-test on proboscis length to test for differences between male and female hawkmoths. We used linear regression to analyze the relationship between the plant’s and pollinator’s fitness and proboscis length.

Statistical analyses were performed in R version 2.3.2.

## 3. Results

### 3.1 The first stage experiment identifies a critical region in flower morphospace where the interests of plants and pollinators are both sensitive to corolla curvature variation

#### 3.1.1 Flowers with gentle curvatures are visited less frequently by hawkmoths

**Fig 3:**
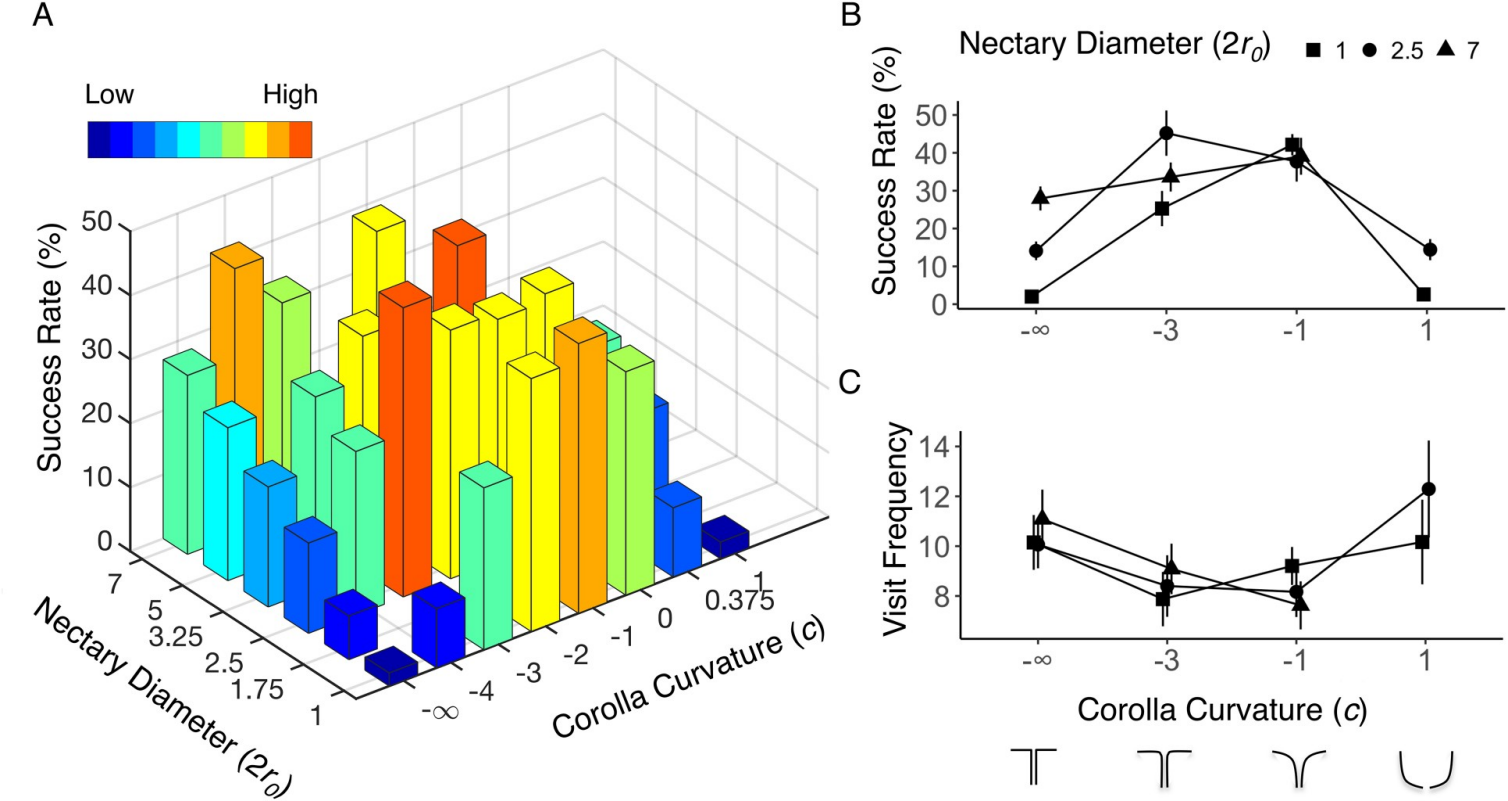
Hawkmoth foraging success rate and flower visitation frequency in the first stage experiment. (A). Hawkmoth foraging success rate across two dimensions of flower morphospace. Panels B and C show some flowers that capture the range of variation along the two morphological axes from the full dataset (S1 Table). (B). Hawkmoth foraging success rate. (C) Flower visitation frequency. Error bars represent ± 1 SEM. The sample size of each flower morphology is in S1 Table.

Flower curvature significantly influences flower visitation frequency (p = 2.56×10^−6^). Flowers with more extreme curvatures (*c* = –∞ or *c* = 1) received up to 45% more visits than the flowers with gentle curvatures (*c* = –1 or *c* = –2) (Fig 3C). Nectary diameter (p = 0.31) and the interaction between nectary diameter and corolla curvature (p = 0.29) have no significant influence on flower visitation frequency.

#### 3.1.2 Hawkmoth foraging success rate is maximized in gently-curved flowers

There is a significant effect on hawkmoth foraging success rate due to variation in corolla curvature (p = 2.00×10^−16^), nectary diameter (p = 7.18×10^−9^), and the interaction between nectary diameter and corolla curvature (p = 3.25×10^−5^). The easiest flower morphology for hawkmoths to exploit (*c* = -3, 2*r*_*0*_ = 2.5) yielded an average foraging success rate of 45% ± 6% standard error of the mean (SEM) while the most difficult one (*c* = -∞, 2*r*_*0*_ = 1) was fed upon with an average success rate of 2% ± 0.7% SEM (Fig 3A and 3B). A peak in foraging success occurs among trumpet-shaped flowers, with the hawkmoth’s performance decreasing steadily toward the extremes of corolla curvature: flat (*c* = -∞,) and bowl-shaped (*c* = 1) flowers (Fig 3B). The difference in foraging success rate between trumpet-shaped flowers and flowers with extreme curvatures is magnified as the nectary diameter decreases (Fig 3B).

#### 3.1.3 Hawkmoth foraging success rate is sensitive to curvature only when the nectary diameter is small

Individually, a large nectary diameter and a trumpet-shaped corolla curvature yield similarly high foraging success rates, at about 40% per foraging trial (Fig 3B). Foraging performance decreases with decreasing nectary diameter. However, poor foraging performance at small nectary diameters (at or below 2.5 mm) can be rescued by the trumpet-like curvature (*c* = -1 and -2) to maximal performance (Fig 3A and 3B).

### 3.2 The second stage experiment reveals non-overlapping fitness optima in both flower corolla curvature and hawkmoth proboscis length

#### 3.2.1 Non-overlapping fitness optima exists with respect to flower corolla curvature variation

**Fig 4:**
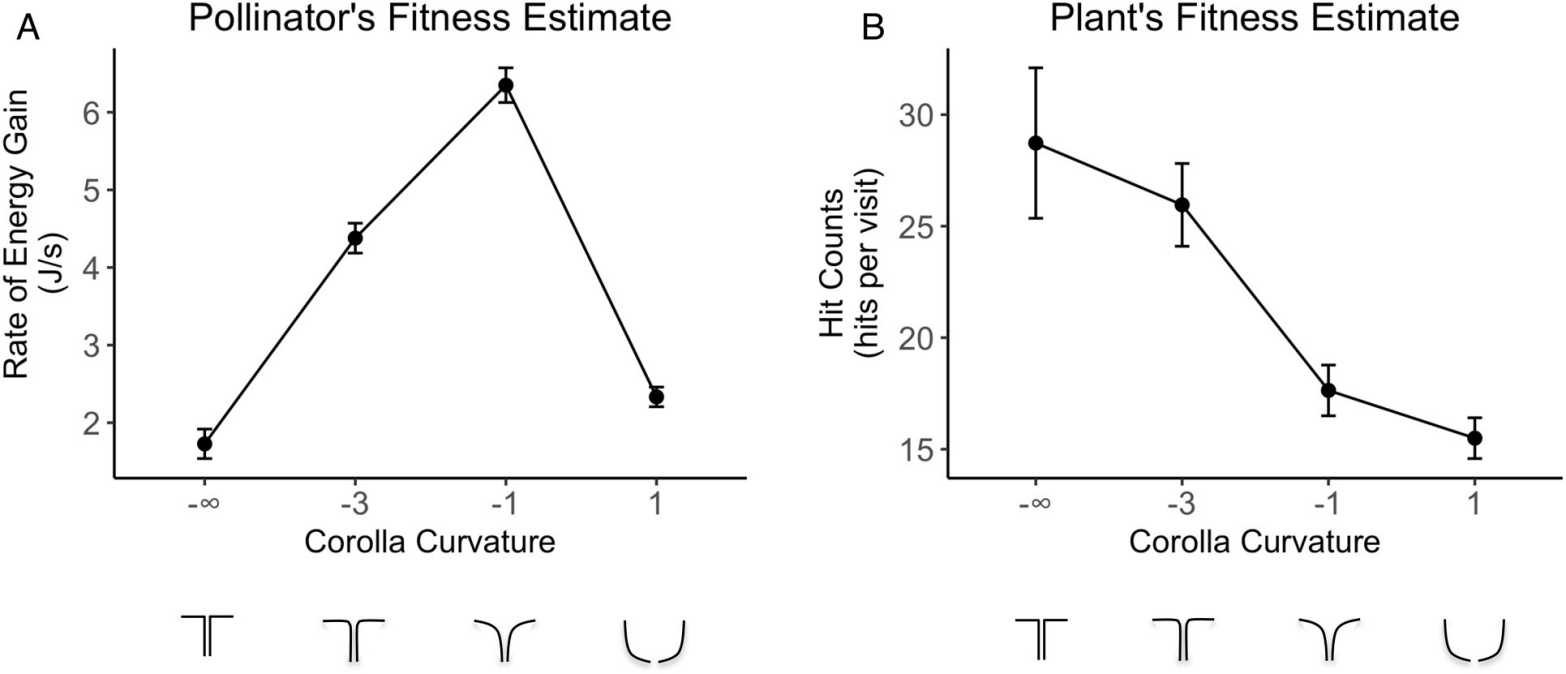
Fitness of pollinator and plant measured by visit *quality*. (A). Hawkmoth pollinator’s fitness; (B). Plant’s fitness. Error bars are ±1 SEM. Sample size (number of visits) of each flower morph is: c = -∞, N = 198; c = -3, N = 428; c = -1, N = 384; c = 1, N = 806.

Corolla curvature significantly influences both the plant’s (p = 1.92×10^−10^) and the pollinator’s fitness (p = 2.00x10^-16^). The hawkmoth’s fitness is maximal for trumpet-shaped flowers (*c* = -1; 6.35 ± 0.22 J/s/visit SEM), and steadily decreases towards the extremes of flat (*c* = −∞; 1.73 ± 0.19 SEM) and bowl-shaped flowers (*c* = 1; 2.33 ± 0.17 SEM), producing a bell-shaped fitness curve (Fig 4A). This result is concordant with the findings from the first stage experiment when we used foraging success rate as a measure of the hawkmoth’s fitness (Fig 3B), and suggests that the influence of corolla curvature on the hawkmoth’s foraging performance is robust to the differences in experimental design and fitness measurement between first stage and second stage experiments.

The plant’s fitness estimate decreases steadily as corolla curvature increases (Fig 4B). The bowl-shaped flower receives the fewest physical contacts (*c* = 1; 15.49 ± 0.91 hits/visit SEM), while the flat flower receives the most contacts (*c* = −∞; 28.73 ± 3.37 SEM) (Fig 4B).

#### 3.2.2 Non-overlapping fitness optima with respect to hawkmoth proboscis length variation

In addition to the engineering approach to morphospace analysis, we took advantage of the natural variation in hawkmoth proboscis length to evaluate its influence on plant and pollinator fitness. There is no significant difference (two-tailed t-test, t = 0.67, p = 0.51) in proboscis length between male (82.19 ± 3.92 mm standard deviation, N = 27) and female (82.77 ± 2.55 standard deviation, N = 31) hawkmoths.

Hawkmoth fitness increased linearly with the increase of proboscis length (F-test, p = 0.0001, S2A Fig), but plant’s fitness decreased linearly with the increase of proboscis length (F-test, p = 0.02, S2B Fig).

## 4. Discussion

Although traditionally the relationship between plants and their pollinators is viewed as mutualistic, they have different requirements from their interaction: efficient pollen transfer for the plant *vs*. efficient energy acquisition for the pollinator. Using a novel combination of engineering technologies (3D printing, artificial flowers instrumented with an IR nectar sensor and an accelerometer contact sensor, automated nectar replenishment, and machine vision) to explore this interaction in flower morphospace, we have shown that these disparate requirements generate divergent fitness optima between plants and pollinators with respect to flower shape (Figs 3B and 3C, 4A and 4B).

As a proof-of-concept exploration, we first examined the pollinator’s fitness in terms of visit *quantity* in a two-dimensional flower morphospace, varying corolla curvature and nectary diameter. We showed that the influence of these two floral morphological features on pollinator performance is non-additive. Flowers with smaller nectary diameters are more difficult for the hawkmoths to exploit, but this effect could be countered by an appropriate corolla curvature (*e*.*g*. trumpet-shaped, *c* = -1) (Fig 3A). The powerful theoretical morphospace paradigm [8] enabled us to study both the effects of single morphological traits and the interactions among them without bias or constraint, and we successfully demonstrated its utility by revealing a previously undiscovered interaction between corolla curvature and nectary diameter.

Corolla curvature is a key trait that significantly influences both flower visitation frequency and foraging success rates by the hawkmoth pollinator. Our second stage experiment used a novel approach to measure visit *quality*; *i*.*e*. effectiveness in the critical region of flower morphospace where hawkmoth pollinator foraging performance is exquisitely sensitive to corolla curvature. We found that there are non-overlapping fitness optima between plants and pollinators with respect to corolla curvature. The pollinator’s fitness peaks at the trumpet shaped flower (*c* = –1), while the plant’s fitness peaks at the flat disc flower (*c* = –∞) (Fig 4A and 4B).

This result can be understood from the mechanical basis of the two disparate activities—nectar acquisition for the pollinator *vs*. pollen transfer for the plant. From the perspective of the pollinator’s fitness, hawkmoths rely on mechanosensory information from the proboscis to locate the nectary in low light conditions [13]. Flower corolla curvature can act as a mechanical guide to assist hawkmoth pollinators in finding the nectary [9]. The flat disc flower (*c* = –∞) and bowl-shaped flower (*c* = 1) probably provide less passive guidance of the proboscis and more ambiguous mechanosensory information about the location of the nectary than does the trumpet shaped corolla (*c* = -1). From the perspective of plant’s fitness, hawkmoths must position their heads closer to the center of flower in order to deal with the more abruptly changing curvature (*c* = -∞ and c = -3), which leads to stronger contacts with the plant’s reproductive parts (anther/stigma) (S2 and S3 Videos). Gently curved flowers (c = -1) and bowl-shaped flowers (c = 1) have wider openings, with greater latitude for the position of the hawkmoth’s head, so the plant’s reproductive parts received fewer contacts. As a result, the intrinsic difference between the requirements of the plant and pollinator generate non-overlapping peaks in the fitness landscape.

The non-overlapping fitness optima in flower morphology motivated us to take advantage of the natural morphological variation in the length of the hawkmoth pollinator’s proboscis. Hawkmoth proboscis length variation has long been suspected to play a key role in flower nectar spur evolution [6], but its effects on the fitness of both plants and pollinators have seldom been explicitly tested (but see [14]). When presented with flowers having an invariant corolla tube length (70 mm, from the flower top plane to the bottom of the nectar reservoir) shorter than the shortest hawkmoth *M*. *sexta* proboscis (72 mm) in our experiment, we found that hawkmoths with longer proboscides are more proficient at nectar feeding, but make fewer contacts with the flower’s reproductive parts, probably because the hawkmoth inserts its proboscis no farther than necessary to obtain nectar [15]. However, the distance from the hawkmoth’s body to the center of the flower is also greater, so the hawkmoth’s body is less likely to contact the flower’s reproductive parts. As a result, a longer proboscis benefits the hawkmoth while harming the plant’s reproductive interests. Intuitively, a longer corolla tube or nectar spur on the flower would have the opposite effect on each party. This result corroborates previous field studies showing an evolutionary conflict between pollinator proboscis length and flower tube length [14,16]. It also lends support for the classical hypothesis that an arms race between plants and pollinators drives the evolution of long proboscides in hawkmoths and correspondingly long corolla tubes and nectar spurs in flowers [17]. Such an arms race can lead to the extreme morphologies typified by Darwin’s orchid and its hawkmoth pollinator, whose nectar spur and proboscis can be up to 45 cm in length [6].

One potential consequence of non-overlapping fitness optima between plants and pollinators is evolutionary diversification. Plant populations with highly variable flower morphologies could diverge by pollinator-mediated assortative mating, with each flower morph having its highest fitness with a “matched” pollinator. Ultimately, this assortative mating could lead to plant speciation by pollinator shift—the origin of new plant species pollinated by different pollinator guilds (*e*.*g*., hawkmoths, bumblebees, hummingbirds, bats) best able to pollinate each alternative flower morph.

We are aware of that certain limitations exist in this artificial flower model approach. For example, in the simplified 3D-printed flower models, some features are not directly comparable to natural flowers, such as petal texture and mechanical properties (PVC plastic *vs*. natural petal tissues), which could impact the efficiency in plant-pollinator interactions. However, our approach serves as an alternative to understand some aspects of plant-pollinator interactions which are difficult or impossible to manipulate or measure in traditional field ecological experiments, such as the details of energy acquisition/expenditure. The combination of 3D printing technologies, electronic sensing, and machine vision has enabled us to rationally design flower morphologies, accurately generate flower models with desired parameters, and automate high-throughput behavioral data collection during plant-pollinator interactions. In the future, field pollination experiments could also benefit from the deployment of such engineered systems, especially for studying night-foraging pollinators, such as hawkmoths and bats.

We can extend our exploration of flower morphospace to other features (*e*.*g*., corolla tube length, petal number and shape, color, scent, texture), and also map the fitness landscapes of animals representing other pollinator guilds to see if our finding is generalizable. If complemented with *in plastico* experimental evolution on artificial flower populations, we could further investigate—in real time—how the divergent selective force exerted by different pollinator guilds drives flower pollination syndrome divergence.

## Supporting information

S1 Fig Line drawing depicting major elements of the first stage experimental arena. (DOCX)

S2 Fig The length of hawkmoth proboscis is an evolutionay conflict between plants and hawkmoths. (DOCX)

S1 Table The full data set of the first stage experiment. (DOCX)

S2 Table The full data set of the second stage experiment. (DOCX)

S1 Video The video recording of two consecutive visits in the second stage experiment under infrared light. (MP4)

S2 Video Hawkmoth visit to a flower with relatively simple morphology to exploit. (MP4)

S3 Video Hawkmoth visit to a flower with relatively difficult morphology to exploit. (MP4)
