# Supplementary material for: Morphospace exploration reveals divergent fitness optima between plants and pollinators
Source: PLoS One. 2019 Mar 13;14(3):e0213029. doi: 10.1371/journal.pone.0213029 (PMC6415803; doi:10.1371/journal.pone.0213029)
Supplement: S1 Fig — (DOCX) [file pone.0213029.s001.docx]

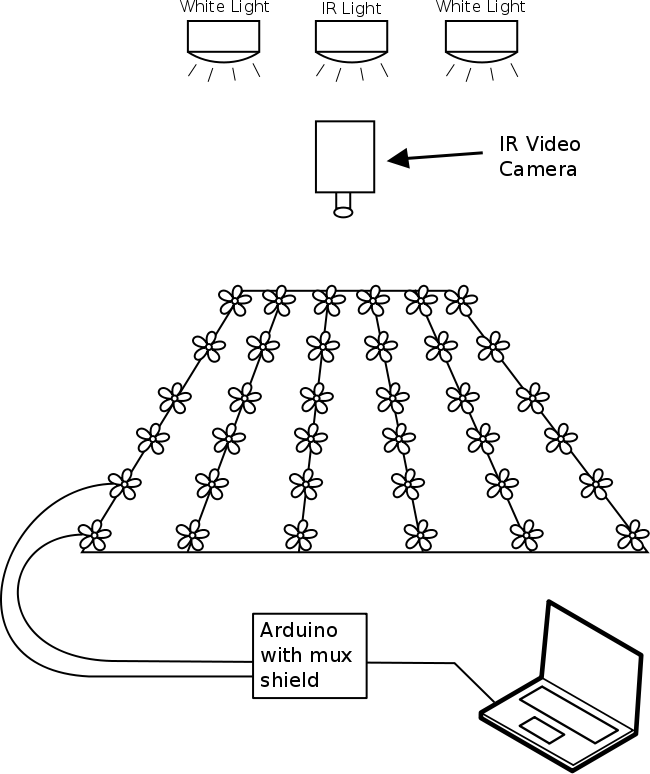


**Figure S1. Line drawing depicting major elements of the first stage experimental arena.** The morphological disparity of the artificial flowers populating the array is not reflected here. Each artificial flower was attached to an infrared sensor to detect when proboscis insertion into the nectary occurred at each flower, and to calculate the duration of each probing event. The signal from each flower was routed to an Arduino® microcontroller. For simplicity, only two of the flowers are shown "wired up" in this graphic.
