# Supplementary material for: Morphospace exploration reveals divergent fitness optima between plants and pollinators
Source: PLoS One. 2019 Mar 13;14(3):e0213029. doi: 10.1371/journal.pone.0213029 (PMC6415803; doi:10.1371/journal.pone.0213029)
Supplement: S2 Fig — (DOCX) [file pone.0213029.s002.docx]

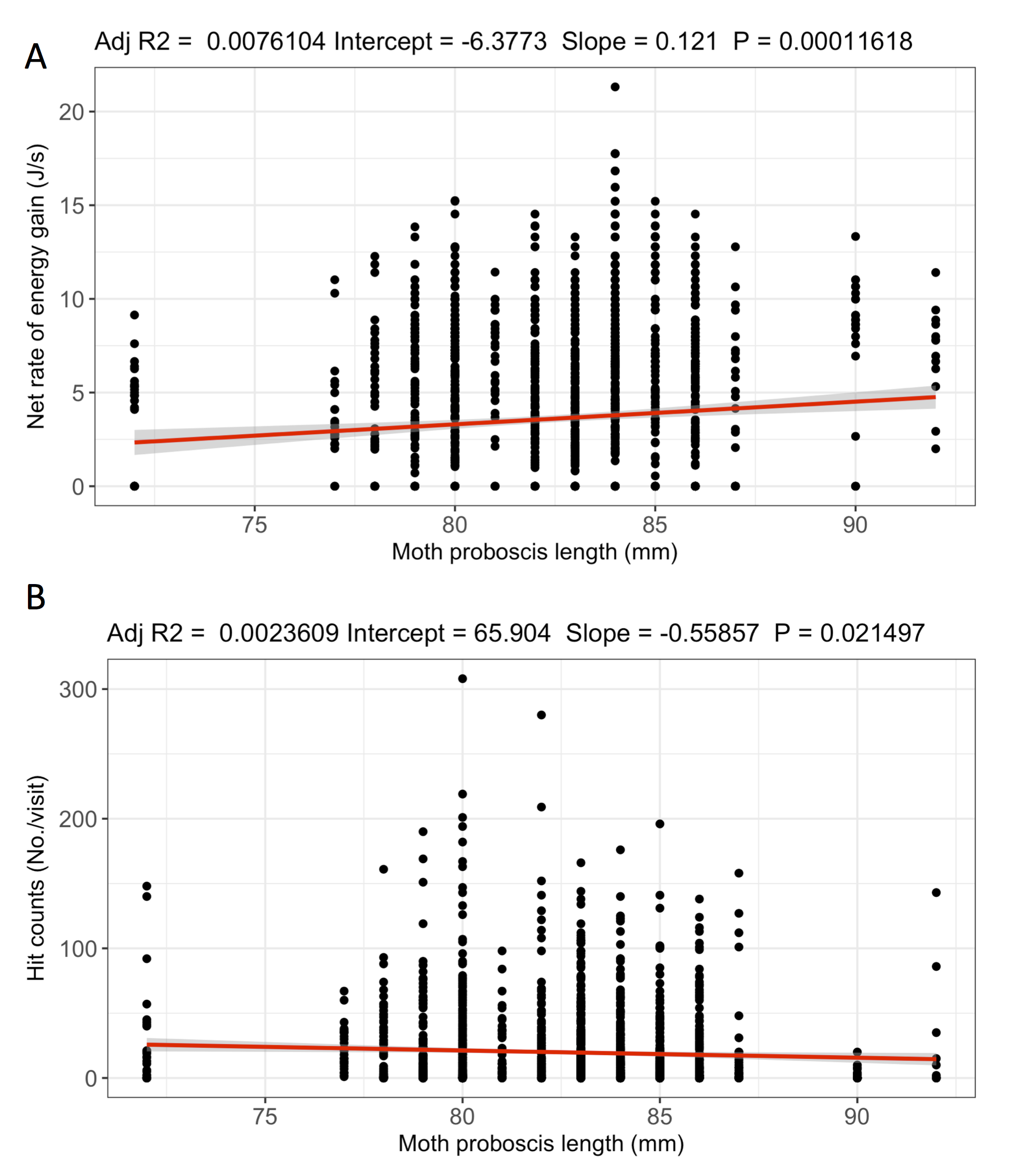


**Figure S2. The length of hawkmoth proboscis is an evolutionay conflict between plants and hawkmoths**.

The data is pooled from all the trials in the second stage experiment. The gray smooth area represents 95% confidence interval for predictions of the linear model (red line). The upper panel (A) shows the positive linear relationship between proboscis length and pollinator’s fitness measured as net rate of energy gain. The lower panel (B) shows the negative relationship between proboscis length and plant’s fitness measured as hit counts per visit.
