# Supplementary material for: Morphospace exploration reveals divergent fitness optima between plants and pollinators
Source: PLoS One. 2019 Mar 13;14(3):e0213029. doi: 10.1371/journal.pone.0213029 (PMC6415803; doi:10.1371/journal.pone.0213029)
Supplement: S1 Table — (DOCX) [file pone.0213029.s003.docx]

**Table S1. The full data set of the first stage experiment**

| Corolla Curvature (c) | Nectary Diameter (2r_0_, mm) | Sample Size | Average Visit Frequency | SEM of Visit Frequency | Average Success Rate (%) | SEM of Success Rate |
| --- | --- | --- | --- | --- | --- | --- |
| -∞ | 1 | 47 | 10.15 | 1.10 | 2.02 | 0.74 |
| -4 | 1 | 24 | 8.08 | 1.42 | 9.22 | 2.58 |
| -3 | 1 | 24 | 7.88 | 1.09 | 25.27 | 4.66 |
| -2 | 1 | 25 | 8.08 | 0.92 | 39.51 | 5.10 |
| -1 | 1 | 73 | 9.21 | 0.76 | 42.14 | 2.81 |
| 0 | 1 | 25 | 11.28 | 1.49 | 34.90 | 4.12 |
| 0.375 | 1 | 24 | 8.79 | 0.98 | 10.78 | 2.69 |
| 1 | 1 | 24 | 10.17 | 1.70 | 2.59 | 1.25 |
| -∞ | 1.75 | 25 | 13.16 | 1.96 | 6.82 | 2.00 |
| -∞ | 2.5 | 47 | 10.06 | 0.95 | 14.09 | 2.47 |
| -4 | 2.5 | 23 | 7.39 | 1.00 | 25.56 | 4.65 |
| -3 | 2.5 | 25 | 8.40 | 1.23 | 45.20 | 5.98 |
| -2 | 2.5 | 24 | 7.42 | 1.13 | 38.85 | 5.20 |
| -1 | 2.5 | 24 | 8.17 | 1.01 | 37.69 | 5.29 |
| 0 | 2.5 | 24 | 7.75 | 1.43 | 38.84 | 5.93 |
| 0.375 | 2.5 | 25 | 12.72 | 1.75 | 27.68 | 3.85 |
| 1 | 2.5 | 24 | 12.29 | 1.95 | 14.42 | 2.80 |
| -∞ | 3.25 | 24 | 11.54 | 1.84 | 18.75 | 3.14 |
| -4 | 3.25 | 25 | 5.56 | 0.83 | 29.99 | 4.69 |
| -3 | 3.25 | 25 | 6.84 | 0.88 | 36.62 | 5.81 |
| -1 | 3.25 | 25 | 6.04 | 0.75 | 45.09 | 5.10 |
| -∞ | 5 | 25 | 10.96 | 1.82 | 23.89 | 3.12 |
| -∞ | 7 | 24 | 11.08 | 1.19 | 27.95 | 3.18 |
| -4 | 7 | 25 | 6.96 | 1.13 | 41.80 | 5.30 |
| -3 | 7 | 23 | 9.09 | 1.01 | 33.61 | 3.83 |
| -1 | 7 | 24 | 7.63 | 0.96 | 39.08 | 4.88 |
