# Supplementary material for: Morphospace exploration reveals divergent fitness optima between plants and pollinators
Source: PLoS One. 2019 Mar 13;14(3):e0213029. doi: 10.1371/journal.pone.0213029 (PMC6415803; doi:10.1371/journal.pone.0213029)
Supplement: S2 Table — (DOCX) [file pone.0213029.s004.docx]

**Table S2. The full data set of the second stage experiment**

| Corolla Curvature (c) | Nectary Diameter (2r_0_, mm) | Sample Size | Average Hit Count | SEM of Hit Count | Averge Visit Time (s) | SEM of Visit Time | Average Rate of Energy Gain (J/s) | SEM of Rate of Energy Gain |
| --- | --- | --- | --- | --- | --- | --- | --- | --- |
| -∞ | 2.5 | 198 | 28.73 | 3.37 | 13.10 | 1.09 | 1.73 | 0.19 |
| -3 | 2.5 | 428 | 25.96 | 1.86 | 12.11 | 0.51 | 4.38 | 0.19 |
| -1 | 2.5 | 384 | 17.64 | 1.14 | 10.51 | 0.42 | 6.35 | 0.22 |
| 1 | 2.5 | 806 | 15.49 | 0.92 | 9.85 | 0.31 | 2.33 | 0.13 |
